# Gaps in governance: protective mechanisms used by nurse leaders when policy and practice are misaligned

**DOI:** 10.1186/s12913-015-0827-y

**Published:** 2015-04-09

**Authors:** Kaye M Knight, Amanda Kenny, Ruth Endacott

**Affiliations:** La Trobe Rural Health School, Bendigo, VIC Australia; Centre for Health and Social Care Innovation, Faculty of Health and Human Sciences, University of Plymouth, Drake Circus, Plymouth UK & Monash University, Melbourne, Australia

**Keywords:** Rural, Nursing, Practice, Empowerment, Clinical governance, Leadership, Telephone

## Abstract

**Background:**

Due to large geographical distances, the telephone is central to enabling rural Australian communities to access care from their local health service. While there is a history of rural nurses providing care via the telephone, it has been a highly controversial practice that is not routinely documented and little is known about how the practice is governed. The lack of knowledge regarding governance extends to the role of Directors of Nursing as clinical leaders charged with the responsibility of ensuring practice safety, quality, regulation and risk management. The purpose of this study was to identify clinical governance processes related to managing telephone presentations, and to explore Directors of Nursing perceptions of processes and clinical practices related to the management of telephone presentations to health services in rural Victoria, Australia.

**Methods:**

Qualitative documentary analysis and semi structured interviews were used in the study to examine the content of health service policies and explore the perceptions of Directors of Nursing in eight rural health services regarding policy content and enactment when people telephone rural health services for care. Participants were purposively selected for their knowledge and leadership role in governance processes and clinical practice. Data from the interviews were analysed using framework analysis. The process of analysis resulted in the identification of five themes.

**Results:**

The majority of policies reviewed provided little guidance for managing telephone presentations. The Directors of Nursing perceived policy content and enactment to be largely inadequate. When organisational structures failed to provide appropriate governance for the context, the Directors of Nursing engaged in protective mechanisms to support rural nurses who manage telephone presentations.

**Conclusions:**

Rural Directors of Nursing employed intuitive behaviours to protect rural nurses practicing within a clinical governance context that is inadequate for the complexities of the environment. Protective mechanisms provided indicators of clinical leadership and governance effectiveness, which may assist rural nurse leaders to strengthen quality and safe care by unlocking the potential of intuitive behaviours. Kanter’s theory of structural power provides a way of conceptualising these protective mechanisms, illustrating how rural nurse leaders enact power.

## Background

Providing leadership to ensure quality and safe clinical outcomes for patients is a core expectation of nurse leaders [1], with clinical governance central to quality and safe care [2]. However, clinical governance systems and processes are primarily developed for medium to large urban healthcare settings and do not accommodate for some of the differences and complexities of the rural healthcare context [3,4]. One such complexity is that nurses receive telephone calls from rural people seeking advice and care yet most telephone presentations are not documented and little is known about how the practice is governed [5].

In Victoria, Australia, small rural health services are expected to manage all urgent care presentations, whether people present in person or via the telephone [6]. Despite the emergence of dedicated telephone nursing services, rural people continue to call their local health service for care [7]. In the 1990s researchers identified Australian rural nurses providing care via the telephone even when clinical governance processes, such as organisational policies and practice guidelines, are absent or inadequate to support the practice [8,9]. No further research into Australian rural nurses providing care via the telephone outside of dedicated telephone triage services has been conducted. However, anecdotally, this situation continues [5], challenging the ability of nurse leaders to lead quality and safe clinical care. Little is known about the clinical governance processes related to telephone care and how rural nurse leaders manage this practice.

Clinical governance has become the cornerstone of healthcare quality and safety [10,11]. Considered an umbrella term [11], clinical governance is defined as “Structures, systems, and standards applying to create a culture, and direct and control clinical activities.” ([11] p119). Organisational structures, systems and standards include: a patient focus, risk management, relevant policies, evidence based practice standards, clinical indicators, regulation, training, monitoring and compliance [2,10,11].

While the concept of clinical governance is well accepted, a gap between the theory of clinical governance and its application in practice has been found in urban [11,12] and rural [13-15] health care settings. Researchers who have explored differences in implementing quality and safety strategies in rural health care settings to that of urban settings, have identified that practical application of clinical governance systems can be problematic due to the complexities of the rural environment. Rural specific barriers to implementing clinical governance include: limited staffing, resources and infrastructure, personnel and patient characteristics [4,13,16]; perceived differences in how quality patient outcomes should be measured in rural health settings [3,17] and; the imposition of urban based governance systems that are a poor fit for rural health services [15,17].

The complexities of rural nursing in Australia, the United States (US) and Canada have been well documented over the past decade and include: professional isolation, the breadth of knowledge required, dual relationships, lack of anonymity, limited access to health resources, ageing nursing workforce, problematic recruitment and retention of medical, nursing and allied health staff, and nursing scope of practice issues [18-23]. Researchers argue that health services have failed to adequately manage these complexities, spawning nursing practices that fall outside clinical governance processes and nurses’ scope of practice [19,24,25]. Providing care via the telephone is one example of a practice that falls outside clinical governance processes [5,9].

Dedicated telephone nursing services are now recognised as a legitimate form of healthcare in Australia [26-28]. However, the provision of care via the telephone by nurses in the absence of, or outside, health service policy is not recognised and is actively discouraged [29]. Despite this, studies have indicated that rural nurses provide care to their local community via the telephone [5], leaving nurses unsupported in this practice [19,25].

The Director of Nursing (DoN), or equivalent, is the most senior nurse in Australian rural health services. As nurse leaders, the DoN is responsible for the strategic management of the nursing workforce, financial management, policy development, clinical governance, and care outcomes at the operational level [30]. Given there is a direct link between the leadership practices of nurse leaders and quality of care outcomes [1], DoNs play a crucial role in facilitating quality and safety in nursing care. However, researchers have found the complexities of rural health care has led to rural nurse leaders allowing practices outside of organisational policy and nurses’ scope of practice to occur [20]. Little is known about how the management of telephone presentations is governed in rural health services. The aim of this study was to examine rural health service policy related to managing telephone presentations and to identify DoN perceptions regarding their health service policy, enactment and factors influencing this enactment.

## Methods

### Research design

Multi-case, embedded case study design [31] was used for this qualitative study. Case study design is recommended where a phenomenon is controversial, occurs in real life and little is known [31]. Each rural health service was considered a ‘case’, and was selected based on shared characteristics including: rural location, types of services offered including Urgent Care services, absence of doctors on staff and, confirmation of telephone presentations by people seeking care. Using multiple cases provided the opportunity to gather multiple sources of data resulting in a more substantial and compelling study. The study incorporated three stages: DoN interviews, health service policy review and registered nurse interviews. The registered nurse stage of this study has been published [32]. This article reports on the findings from the DoN interviews and review of their health service policy.

The aims of this stage of the study were to: identify clinical governance processes related to managing telephone presentations, and to explore Directors of Nursing perceptions of processes and clinical practices related to the management of telephone presentations to health services in rural Victoria, Australia.

### Setting and sample

This study was conducted in eight small rural health services in Victoria, Australia (see Table 1). Small rural health services are situated outside metropolitan areas [22], and generally provide residential aged care, primary health care, acute care and urgent care services [6]. All Victorian rural health services are within a 30–120 minute drive to a larger regional health service. There are generally no doctors on staff; however, nurses have on-call access to community based general medical practitioners (GPs) who provide treatment instructions either face to face or via the telephone. People present to these health services, in person and via the telephone, and there is an expectation that these presentations will be managed initially in the rural health service [6].

**Table 1:** **Health service profiles**

**Health service**	**Services offered**	**No. of acute care beds**	**Approximate travel time by car to regional health service**	**After hours GP access**
**A**	Residential aged care, acute care, primary care, urgent care.	<35	90 mins	On call but may not be in same town
**B**	Residential aged care, acute care, primary care, urgent care.	<5	110 mins	On call
**C**	Residential aged care, acute care, primary care, urgent care.	<35	35 mins	On call
**D**	Residential aged care, acute care, primary care, urgent care.	<25	70 mins	On call
**E**	Residential aged care, acute care, primary care, urgent care.	<10	90 mins	On call but may not be in same town
**F**	Residential aged care, acute care, primary care, urgent care.	<20	50 mins	On call but may not be in same town
**G**	Residential aged care, acute care, primary care, urgent care.	<10	40 mins	On call but may not be in same town
**H**	Residential aged care, acute care, primary care, urgent care.	<30	40 mins	On call

The sample in this study was registered nurses holding the nursing leadership position of DoN, or equivalent, in a Victorian small rural health service, with a minimum of six months in their current position. As the most senior nurse in the health service, DoNs were selected purposively [31] for their knowledge and leadership role related to organisational governance processes and clinical practice. Eight DoNs agreed to participate in the study.

### Data collection

Telephone interviews were conducted in 2009 as the first stage of a three stage study that was completed in 2012. During the study period researchers checked for policy and workforce changes at the study sites with no changes identified. Interviews were scheduled at a time that suited participants, were approximately 30 minutes in length and audio taped with permission. A semi structured interview approach enabled the DoNs to share perceptions and opinions [31]. The interview schedule included questions related to the management of telephone presentations such as: organisational policy, satisfaction with policy content and enactment, and perceptions of the factors impacting on policy enactment. DoNs from the five health services that had policies in place agreed to provide a copy of the policy for analysis in this study.

### Ethical approval

La Trobe University Faculty Human Ethics Committee approved this study. Participation was voluntary and strategies to ensure anonymity were implemented including de-identification of health service policies and participant quotes.

### Data analysis

Two types of data analysis were used in this study. Qualitative content analysis [33] was used to analyse health service policies. Framework analysis [34] was used to analyse data collected via the DoN interviews.

Existing documents, such as health service policies, reflect the context in which they were developed [35]. As contextually relevant sources of information, documents are recommended sources of data in case study design [31]. Qualitative content analysis enabled the identification of common elements and unique expectations regarding governance and nursing practice related to telephone presentations across the study sites [33].

An approach relevant for policy based qualitative research, framework analysis consists of five stages: familiarisation, identify a thematic framework, indexing, charting and, mapping and interpretation. These linked stages provided transparency in the data analysis process of the DoN interviews and enabled the researcher to move forward and backward across the data refining the emerging themes [36].

Familiarisation was achieved through listening to the audiotape of the interviews, transcribing the interviews, and reading the transcripts several times to identify initial themes. The semi structured interview schedule provided a number of ‘a priori’ themes. New themes that emerged were identified as the DoNs discussed the context of their health service, staff and rural community.

Identified themes drawn from the health service policy analysis and interview data were labeled, given a numerical annotation and used to index each transcript. Themes were refined through the indexing process and chunks of data were arranged according to the refined thematic labels and charted within a matrix against the unique identifier used for each participant. This enabled data re-immersion to check that the interpretations remained true to the data. The charts facilitated the interpretation of the data to determine key themes and concepts, and the relationships between the themes.

### Establishing rigor

Lincoln & Guba’s [35] criteria for establishing trustworthiness: credibility, dependability, confirmability and transferability, were applied in this study. Credibility was achieved by sending interview transcripts to the DoNs for checking, with no amendments requested. Strategies to achieve dependability included: the use of a structured interview schedule, audio recording and transcription of the interviews, the application of framework analysis that outlines the analysis process and, agreement of themes reached by all researchers. The use of a detailed research protocol outlining research steps, a structured interview schedule and the recruitment of DoNs from multiple sites achieved confirmability. A detailed description of the DoN perceptions regarding policy enactment may facilitate transferability to other contexts.

## Results

Data analysis revealed five themes: Attempting governance, Being local, Role of experience, Managing limited resources and Protective mechanisms.

### Attempting governance

This theme presents the notion that governance measures do not necessarily align with the practice context. Health service policy content and the perceptions of the DoNs regarding the adequacy of health service policy are presented together, highlighting governance attempts rather than achievement.

Of the eight health services included in the study, only five had current policies related to telephone presentations. Two of the five policies provided clear instructions related to the management of telephone presentations. Two policies addressed the broader concept of management of after hours emergency presentations, providing only scant details on how to manage the telephone presentations. The remaining policy included a lengthy legal statement warning nurses not to provide assistance over the telephone unless the caller had a history of admission to the health service. The areas of guidance in health service policies are summarised in Table 2

**Table 2:** **Health service policy guidance provided and DoN satisfaction**

**Health service & DoN perceived frequency of telephone presentations**	**Need to document calls**	**Clinical protocols**	**Quality evaluation**	**Level of nurse managing telephone presentations**	**DoN satisfaction with adequacy of policy**
**A Regularly**	Yes	Yes	Yes	Senior registered nurse	Satisfied with content
**B Frequently**	No Policy				Concerned there is no policy
**C Frequently**	Yes	No	No	Registered nurse with + 1 yr experience	Partially satisfied
**D Occasionally**	Yes	No	No	Senior registered nurse	Not satisfied
**E Regularly**	Yes	Yes	No	Registered nurse with + 3 yrs experience	Satisfied
**F Regularly**	Yes	No	No	Registered nurse	Partially satisfied
**G Occasionally**	No policy				Concerned there is no policy
**H Regularly**	No policy				Old policy removed as not reflective of practice

.

All policies deemed management of the telephone presentations to be the responsibility of the registered nurse and referred to a requirement for telephone presentations to be documented. Health services A, C & D specified that each record includes; date, time, name of caller, contact details, subject of call, description of concern, advice given and name of staff member. The other policies did not specify any details. The use of clinical protocols were included in two policies (A & E), both indicating that protocols were to be used to guide the questioning process and assist the registered nurse in providing appropriate information to the caller. One policy referred to the need for evaluation of quality; however, no specific processes were provided.

All DoNs confirmed that people presented to their health service via the telephone seeking care and that nurses provided care, reinforcing the need for a system to support the practice:

*I know we have this thing about nurses aren’t to give advice over the telephone, but the reality is – in the country you do, and it’s better to control that. (E/DoN)*

Five participants indicated their health services had a current policy related to telephone presentations. Satisfaction with policy content was mixed. The two participants from the health services that had clear policies, and promoted the use of evidence based practice protocols, were the most satisfied with the content. The others expressed a desire to introduce new policies or evidence based practice protocols, as their policies were no longer considered adequate for the practice context:

*I looked at it* [the old policy] *and thought this is not detailed enough for our staff. (H/DoN)*

Although all policies stated the need for documentation, only three participants confirmed there was a system of permanent documentation in place. While four participants raised the issue of legal risk related to telephone presentations and the importance of having the policies to manage risk, none of the health services had active quality improvement processes related to telephone presentations in place. In general, perceived indicators of quality were related to the absence of complaints or adverse outcomes:

*I haven’t had any complaints raised about inappropriate triage on the phone. So from an outcome perspective that hasn’t been a concern. (H/DoN)*

Overall, participants were not satisfied with the level of policy enactment. None could confirm that all calls were documented, and there was a reliance on first-hand knowledge to determine policy enactment and quality in service provision:

*Because I am now a bit more removed from the floor…I’m sure there’s some things that go on that I don’t know about sometimes. (E/DoN)*

Without comprehensive processes around the practice, participants were unclear about how nurses managed the calls. The perceived care nurses were providing included: advising callers to present to the health service to be seen; assessing the call and in an emergency advising them to call an ambulance and; providing *‘motherly’* (E/DoN) advice for low emergency situations.

### Being Local

Despite the availability of dedicated telephone triage services, such as the government funded ‘Nurse on Call’, all participants confirmed that local people continue to present to their local health service via the telephone. This occurred even when there was a telephone message providing an option to be automatically transferred to a dedicated telephone triage service:

*I know that some people will still press the option to speak with the staff. (A/DoN)*

The perception and expectation that this would result in more personalised care was considered a key driver behind this behaviour:

*Look, the community knows their local health service and it’s the first place they want to ring. They know and they trust the nurses that live and work in their community. (C/DoN)*

Participants indicated that a desire to help people within their local community was the nurses’ motivation to provide assistance: ‘…*it is their community and they want to be able to help them.’ (A/DoN)*. Nurses also used their understanding of the local community to provide appropriate assistance for the specific needs of the caller:

*Because of the remoteness of it, the nurses…are more keen to say ‘bring the child in’ or ‘come in’ or ‘call an ambulance and come in’. (B/DoN)*

### Role of experience

All participants suggested that the use of past experience was an important element when managing telephone presentations. They indicated experienced, mature nurses were more willing and able to manage the calls. Participants spoke of the knowing that came with experience and maturity. Participants used terms such as ‘gut-feeling’ (F/DoN), ‘common sense’ (C/DoN) and ‘judgment call’ (D/DoN) to illustrate the use of this knowing and the importance of preserving this element:

*By and large, common sense rules and you don’t want to sabotage that, you want to support that. (C/DoN)*

Confidence was perceived as a key factor with participants suggesting the more confident the nurse felt, the more likely they were to give advice. However, when a nurse lacked confidence, irrespective of experience or maturity, the nurse would advise the caller to present to the health service for face-to-face assessment:

*Some nurses would say – bring them in anyway – because they’re too scared to make a decision over the phone. (F/DoN)*

### Managing limited resources

Several participants identified workplace factors that affect the approach to managing telephone presentations. These factors were resource based and included: irregular workforce, geographical spread of staff, and workload. Participants all identified that the management of telephone presentations was an additional, unaccounted workload for nurses. With the majority of participants indicating that telephone presentations occurred regularly or frequently, and the bulk of presentations occurring after hours, this extra workload had the potential to be significant.

Limited access to GPs after hours for unscheduled health care needs was identified as another significant factor that influenced the need for nurses to manage telephone presentations. As a direct result of this, three health services had introduced systems to manage GPs after hours workloads:

*Mostly* [the after hours policy] *is driven around trying to manage the doctors* [workload] *rather than managing the patients that come in. (F/DoN)*

A participant articulated the reason for the need to manage the GPs workload:

*You don’t want to wear your GP out. (H/DoN)*

### Protective mechanisms

All participants perceived that nurses deviated from health service policy when managing telephone presentations. One participant suggested that nurses might not follow the policy if the nurse knows the caller and has a desire to help that person. Another participant’s suggestion for this deviation related to the unique context:

*Some of the problems that come up don’t fit in the protocol. (E/DoN)*

The nurses’ use of knowledge and experience, their propensity to assist the local community, and limited GP resources presented a dilemma for DoNs. In general, comments reflected a tension between the need for policy compliance and meeting local need for service. There was a protective tone to these comments as participants attempted to justify the nurses’ use of experience and local knowledge when overriding the policy:

*Some of them have been here for forty years and they’ve got heaps of experience and heaps of knowledge, so the advice they give out is fine, but it’s just that they don’t necessarily follow the protocol. (E/DoN)*

Documentation was a key area of non-compliance. Factors impacting on completion of documentation included: perceived seriousness of a call, a preference to report calls verbally, confusion about whether to document the telephone presentation and conflict with clinical workload:

*…they might be anywhere* (in the ward) *when they answer the phone and they don’t have the book in front of them. (H/DoN)*

## Discussion

In this study, data analysis yielded two important findings: first, presence of policy does not guarantee practice governance; and second, DoNs employed protective mechanisms when organisational structures failed to provide appropriate governance for the context.

Variation and inadequacy of the majority of policies reviewed, and a lack of documentation, auditing and monitoring, contrasts with the need for clinical governance systems and structures to be based on relevant, consistent and realistic criteria [2]. This study confirms the findings of earlier studies that care via the telephone continues to be provided outside of organizational policy and processes [5,8,9]. The findings contrast with the international telephone triage literature where authors recommend effective clinical governance processes including [37-40]: organisational policy, evidence based practice protocols/guidelines, specialist preparation of the experienced nurse, documentation and, feedback and quality assurance processes.

While these requirements are foundational elements of quality clinical care [2,10,11], the long-standing controversy and lack of acknowledgement surrounding the practice of providing care via the telephone [5] may have contributed to the absence of consistent clinical governance processes.

The DoNs in this study used mechanisms to protect the actions and non-compliance of the nurses. While the use of protective mechanism by DoNs in this study contrasts with the expectations of clinical care leaders [41,42], the behaviour has been identified previously [43]. The requirement for the DoNs to lead governance processes, while supporting nursing staff to manage the challenges of rural nursing practice, creates a tension. Jasper & Crossan [44] argue this tension is the result of management and nursing values conflicting. Another possibility is a tension between the elements of structural empowerment.

As a nurse leader role, the rural DoNs in this study have authority, visibility, influence and informal alliances [45,46]. However, the organisational structures to support the DoNs ability *‘to get things done’* [46] were lacking. This situation may have impacted upon the behaviour of the DoNs in this study, resulting in protective mechanisms to achieve an outcome. Considered adaptive behaviours to manage the limitations of the system [46], it is unclear whether the protective mechanisms are related to the DoNs’ level of power or powerlessness within the organisation.

According to Kanter [46] structurally empowered people are likely to build alliances with others. In this study, the DoNs’ support for nurses to manage telephone presentations was linked to meeting the healthcare needs of the local community, whilst minimizing after hours workload of the local GPs. Their tendency to protect policy deviations was largely due to the increased nursing workload, lack of resources and the complexities that the conflicting needs generate. The DoNs’ behaviour supported multiple alliances, reinforcing their informal sources of power [46]. However, it also created a silence around policy inadequacies and policy deviations. The lack of auditing and monitoring of practice further concealed the inadequacies and deviations and diverted scrutiny of the local practices. Kanter [46] identifies this behaviour as territorialism and a response to powerlessness.

The DoNs’ descriptions of the nurses’ use of experience and contextual understanding in decision-making fit with the notion of ‘naturalistic decision making’ [43]. There is argument in the rural nursing [22,24,47] and telephone triage literature [38,39,48,49], supporting the application of nurses’ clinical judgment in order to provide safe and appropriate care. While this behaviour challenges telephone nursing governance processes [50], rural specific issues such as: limited access to health resources, dual relationships, geographic isolation and health seeking behaviours of rural people [19,20,22,32], means evidence based practice guidelines and policies do not always fit the situation [43,51]. Researchers argue nurses using contextual knowledge and experience in providing care is an appropriate approach to evidence based nursing [52]. Providing staff with autonomy, greater freedom to use their own discretion, and opportunities to take risks in the workplace are indicators of empowered leadership [46]. However, Kanter [46] also argues that leaders experiencing powerlessness may seek to enhance structural power by allowing selective non-compliance with rules and securing informal alliances with staff through favoritism when it comes to non-compliance.

DoN concerns regarding how telephone presentations were managed were primarily based on a lack of confidence in the policy, and detachment from the clinical area. As a result the DoNs were unsure about how the practice is conducted and the lack of auditing reinforced this lack of knowledge. This concern reflects a desire for control and supervision while avoiding practice monitoring and follow-up, and may be a response to powerlessness [46]. Kanter [46] suggests leaders may be fearful of confronting staff about problematic work practices due to a dependency on staff to achieve results. Recruitment and retention of nurses is a major concern in rural health services [22] and may influence rural nurse leaders decisions to address practices that fall outside organisational policies and processes.

While the structural empowerment of the DoNs in this study cannot be determined, these nurse leaders implemented adaptive behaviours designed to manage the inadequate organisational processes, limitations and complexities of the rural context [46]. The protective mechanisms identified in this study are intuitive behaviours [53] that highlight local cultural elements and complexities within the organisational structures [12]. Management of rural complexities related to governance can be improved by utlising local knowledge of what does and doesn’t work [15] and finding innovative solutions through new ways of thinking [16].

Protective mechanisms have the potential to be important indicators of rural nurse leader empowerment and quality and safe care. The degree to which such mechanisms are engaged could be a cue to the level of structural empowerment of the nurse leader and, the effectiveness of organisational structures in supporting quality and safety in rural nursing care.

### Limitations

The small sample size is a limitation of this study, however, Sandelowski [54] argues that qualitative samples should not be judged on size alone. Rather, it is about attaining the depth of information required to achieve an adequate description [54]. No males DONs participated in the study and therefore the male perspective is not included. The controversial nature of the topic, and risk of damaging information related to organisational governance processes and practice, may have resulted in participants filtering responses. Despite these limitations, the information gained elicited a depth of description that achieved the study purpose. A strength of the study is the elucidation of mechanisms employed by rural nurse leaders when policy and practice are misaligned. The findings could have relevance to other rural health services experiencing misalignment of policy and practice and may be transferable to these settings.

## Conclusions

Consistent policies and clinical governance processes related to telephone presentations to rural health services need to be further developed to accommodate the complexities of the rural context to better support nursing practice and enhance quality and safety in rural health care. A larger research study into the use of protective mechanisms by rural nurse leaders is required to determine the significance and meaning of such mechanisms in rural clinical leadership. The notion of protective mechanisms as indicators of structural empowerment and effective clinical governance requires further discussion and conceptualisation to determine their potential to improve care quality, clinical safety and clinical leadership in rural health.

The known complexities of rural health and the difficulties with implementing clinical governance in rural health care settings, impacts on rural nurse leaders capacity to lead quality and safety outcomes in care. While the provision of care via the telephone outside of clinical governance processes is a product of this situation, it is also a microcosm of clinical leadership and governance in rural healthcare. This microcosm provides insights into local cultural elements that impact on clinical governance and the structural empowerment of rural nurse leaders.
